# Supplementary material for: LTA4H extensively associates with mRNAs and lncRNAs indicative of its novel regulatory targets
Source: PeerJ. 2023 Mar 10;11:e14875. doi: 10.7717/peerj.14875 (PMC10010175; doi:10.7717/peerj.14875)
Supplement: Table S1 — (1) Raw data: the number of original sequences transformed from the original image data obtained by sequencing through base calling; (2) Clean reads: the raw reads are stripped of the adapter sequence, and the number of valid sequences obtained after low-quality bases is used for subsequent analysis; (3) Clean Per: the proportion of clean reads in raw reads; (4) Raw base: the count the number of bases it contains, based on the number and length of raw reads, in G; (5) Clean base: according to the number and length of clean reads, count the number of bases it contains, in G; (6) Base Per: the proportion of Clean base to Raw base; (7) Unique tag: unique tag, the number of non-repeating reads and its proportion of clean reads; (8) Q20: Proportion of bases whose sequencing error rate is less than 1%; (9) Q30: Proportion of bases whose sequencing error rate is less than 0.1%; (10) GC: the percentage of GC bases in the total number of bases; (11) DUP: duplication level. The ratio of duplicate reads to total reads. [file peerj-11-14875-s002.docx]

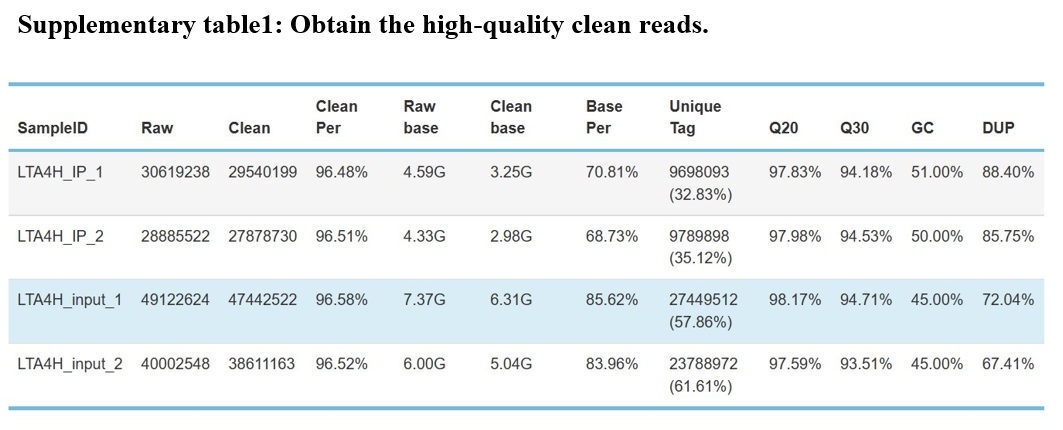


**Table S1. The form shows the quality control of our iRIP-sequence is good.** (1) Raw data: the number of original sequences transformed from the original image data obtained by sequencing through base calling; (2) Clean reads: the raw reads are stripped of the adapter sequence, and the number of valid sequences obtained after low-quality bases is used for subsequent analysis; (3) Clean Per: the proportion of clean reads in raw reads; (4) Raw base: the count the number of bases it contains, based on the number and length of raw reads, in G; (5) Clean base: according to the number and length of clean reads, count the number of bases it contains, in G; (6) Base Per: the proportion of Clean base to Raw base; (7) Unique tag: unique tag, the number of non-repeating reads and its proportion of clean reads; (8) Q20: Proportion of bases whose sequencing error rate is less than 1%; (9) Q30: Proportion of bases whose sequencing error rate is less than 0.1%; (10) GC: the percentage of GC bases in the total number of bases; (11) DUP: duplication level. The ratio of duplicate reads to total reads.
