# Supplementary material for: LTA4H extensively associates with mRNAs and lncRNAs indicative of its novel regulatory targets
Source: PeerJ. 2023 Mar 10;11:e14875. doi: 10.7717/peerj.14875 (PMC10010175; doi:10.7717/peerj.14875)
Supplement: Table S2 — (1) Total reads: input data or clean reads, the number of filtered reads after sequencing sequence; (2) Total mapped: the number of sequences that can be mapped to the genome; (3) Total Uniquely mapped: The number of reads uniquely located on the reference genome and their proportion in total mapped reads. (4) Total Multiple mapped: The number of reads with multiple locations on the reference genome and their proportion in total mapped reads. (5) Splice reads: statistics of sequencing sequences (also known as Junction reads) that are segmented to two exons; (6) Non splice reads: whole-segment comparison of sequencing sequences to exons; (7) rmdup reads: remove duplicate reads, remove the PCR duplication reads, that is, the total number of reads counted after all the repeated reads are counted only once, and their proportion in unique mapped reads; (8) sense reads: reads on the sense chain; (9) antisense reads: reads matched to the antisense chain. [file peerj-11-14875-s003.docx]

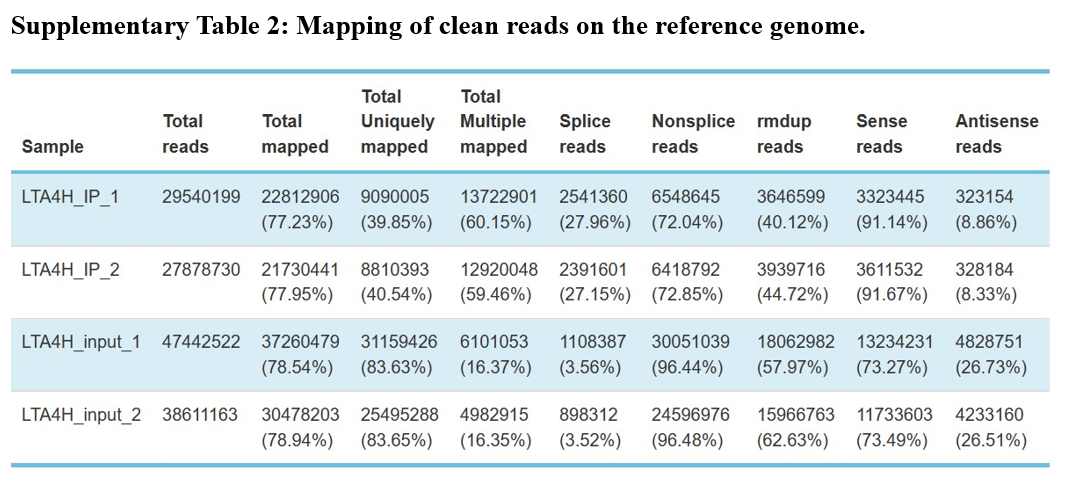


**Table S2. Mapping of clean reads on the reference genome.** (1) Total reads: input data or clean reads, the number of filtered reads after sequencing sequence; (2) Total mapped: the number of sequences that can be mapped to the genome; (3) Total Uniquely mapped: The number of reads uniquely located on the reference genome and their proportion in total mapped reads. (4) Total Multiple mapped: The number of reads with multiple locations on the reference genome and their proportion in total mapped reads. (5) Splice reads: statistics of sequencing sequences (also known as Junction reads) that are segmented to two exons; (6) Non splice reads: whole-segment comparison of sequencing sequences to exons; (7) rmdup reads: remove duplicate reads, remove the PCR duplication reads, that is, the total number of reads counted after all the repeated reads are counted only once, and their proportion in unique mapped reads; (8) sense reads: reads on the sense chain; (9) antisense reads: reads matched to the antisense chain.
