# Supplementary material for: LTA4H extensively associates with mRNAs and lncRNAs indicative of its novel regulatory targets
Source: PeerJ. 2023 Mar 10;11:e14875. doi: 10.7717/peerj.14875 (PMC10010175; doi:10.7717/peerj.14875)
Supplement: File S2 [file peerj-11-14875-s005.zip › Raw data files/Motif_results/ablife/LTA4H_IP_1_vs_LTA4H_input_1/homerResults.html]

.// - Homer de novo Motif Results


# Homer *de novo* Motif Results (.//)

Known Motif Enrichment Results  
Gene Ontology Enrichment Results  
If Homer is having trouble matching a motif to a known motif, try copy/pasting the matrix file into
STAMP  
More information on motif finding results: HOMER
| Description of Results
| Tips
  
Total target sequences = 80683  
Total background sequences = 720379  
\* - possible false positive  

|  |  |  |  |  |  |  |  |  |
| --- | --- | --- | --- | --- | --- | --- | --- | --- |
| Rank | Motif | P-value | log P-pvalue | % of Targets | % of Background | STD(Bg STD) | Best Match/Details | Motif File |
| 1 |  | 1e-717 | -1.652e+03 | 43.44% | 33.18% | 163.6bp (151.2bp) | hsa-miR-4278 MIMAT0016910 Homo sapiens miR-4278 Targets (miRBase)(0.655) More Information | Similar Motifs Found | motif file (matrix) |
| 2 |  | 1e-556 | -1.281e+03 | 35.07% | 26.52% | 161.6bp (166.7bp) | hsa-miR-3183 MIMAT0015063 Homo sapiens miR-3183 Targets (miRBase)(0.700) More Information | Similar Motifs Found | motif file (matrix) |
| 3 |  | 1e-489 | -1.128e+03 | 46.87% | 38.21% | 158.3bp (159.7bp) | hsa-miR-4297 MIMAT0016846 Homo sapiens miR-4297 Targets (miRBase)(0.758) More Information | Similar Motifs Found | motif file (matrix) |
| 4 |  | 1e-443 | -1.020e+03 | 55.17% | 46.81% | 164.3bp (153.6bp) | hsa-miR-4534 MIMAT0019073 Homo sapiens miR-4534 Targets (miRBase)(0.634) More Information | Similar Motifs Found | motif file (matrix) |
| 5 |  | 1e-435 | -1.004e+03 | 37.28% | 29.52% | 175.8bp (144.4bp) | hsa-miR-609 MIMAT0003277 Homo sapiens miR-609 Targets (miRBase)(0.575) More Information | Similar Motifs Found | motif file (matrix) |
| 6 |  | 1e-433 | -9.980e+02 | 20.41% | 14.28% | 168.0bp (150.4bp) | hsa-miR-497\* MIMAT0004768 Homo sapiens miR-497\* Targets (miRBase)(0.650) More Information | Similar Motifs Found | motif file (matrix) |
| 7 |  | 1e-426 | -9.811e+02 | 64.01% | 55.94% | 157.2bp (151.6bp) | hsa-miR-517c MIMAT0002866 Homo sapiens miR-517c Targets (miRBase)(0.656) More Information | Similar Motifs Found | motif file (matrix) |
| 8 |  | 1e-421 | -9.694e+02 | 61.09% | 53.00% | 155.3bp (161.3bp) | hsa-miR-503 MIMAT0002874 Homo sapiens miR-503 Targets (miRBase)(0.631) More Information | Similar Motifs Found | motif file (matrix) |
| 9 |  | 1e-381 | -8.783e+02 | 35.08% | 27.94% | 165.9bp (147.2bp) | hsa-miR-550b MIMAT0018445 Homo sapiens miR-550b Targets (miRBase)(0.719) More Information | Similar Motifs Found | motif file (matrix) |
| 10 |  | 1e-366 | -8.437e+02 | 47.67% | 40.14% | 154.9bp (159.4bp) | hsa-miR-3673 MIMAT0018096 Homo sapiens miR-3673 Targets (miRBase)(0.794) More Information | Similar Motifs Found | motif file (matrix) |
| 11 |  | 1e-343 | -7.903e+02 | 31.17% | 24.64% | 161.5bp (146.6bp) | hsa-miR-4304 MIMAT0016854 Homo sapiens miR-4304 Targets (miRBase)(0.724) More Information | Similar Motifs Found | motif file (matrix) |
| 12 |  | 1e-328 | -7.569e+02 | 66.95% | 59.99% | 169.4bp (147.2bp) | hsa-miR-4275 MIMAT0016905 Homo sapiens miR-4275 Targets (miRBase)(0.631) More Information | Similar Motifs Found | motif file (matrix) |
| 13 |  | 1e-323 | -7.458e+02 | 55.17% | 48.03% | 163.4bp (147.5bp) | hsa-miR-3189-3p MIMAT0015071 Homo sapiens miR-3189-3p Targets (miRBase)(0.700) More Information | Similar Motifs Found | motif file (matrix) |
| 14 |  | 1e-316 | -7.280e+02 | 37.52% | 30.86% | 168.9bp (148.1bp) | hsa-miR-3064-5p MIMAT0019864 Homo sapiens miR-3064-5p Targets (miRBase)(0.785) More Information | Similar Motifs Found | motif file (matrix) |
| 15 |  | 1e-289 | -6.663e+02 | 50.45% | 43.72% | 168.2bp (139.7bp) | hsa-miR-4261 MIMAT0016890 Homo sapiens miR-4261 Targets (miRBase)(0.750) More Information | Similar Motifs Found | motif file (matrix) |
| 16 |  | 1e-281 | -6.478e+02 | 2.89% | 1.16% | 148.6bp (153.7bp) | hsa-miR-4455 MIMAT0018977 Homo sapiens miR-4455 Targets (miRBase)(0.744) More Information | Similar Motifs Found | motif file (matrix) |
| 17 |  | 1e-262 | -6.037e+02 | 41.59% | 35.37% | 163.3bp (151.0bp) | hsa-miR-3145-5p MIMAT0019205 Homo sapiens miR-3145-5p Targets (miRBase)(0.702) More Information | Similar Motifs Found | motif file (matrix) |
| 18 |  | 1e-255 | -5.881e+02 | 68.50% | 62.44% | 168.9bp (135.5bp) | hsa-miR-4760-5p MIMAT0019906 Homo sapiens miR-4760-5p Targets (miRBase)(0.688) More Information | Similar Motifs Found | motif file (matrix) |
| 19 |  | 1e-254 | -5.860e+02 | 77.78% | 72.25% | 159.5bp (147.1bp) | hsa-miR-3669 MIMAT0018092 Homo sapiens miR-3669 Targets (miRBase)(0.613) More Information | Similar Motifs Found | motif file (matrix) |
| 20 |  | 1e-225 | -5.184e+02 | 70.01% | 64.39% | 159.9bp (154.2bp) | hsa-miR-2681 MIMAT0013516 Homo sapiens miR-2681 Targets (miRBase)(0.711) More Information | Similar Motifs Found | motif file (matrix) |
| 21 |  | 1e-220 | -5.083e+02 | 67.61% | 61.96% | 167.1bp (146.2bp) | hsa-miR-144\* MIMAT0004600 Homo sapiens miR-144\* Targets (miRBase)(0.798) More Information | Similar Motifs Found | motif file (matrix) |
| 22 |  | 1e-213 | -4.926e+02 | 16.41% | 12.41% | 146.2bp (154.6bp) | hsa-miR-425\* MIMAT0001343 Homo sapiens miR-425\* Targets (miRBase)(0.693) More Information | Similar Motifs Found | motif file (matrix) |
| 23 |  | 1e-207 | -4.776e+02 | 13.44% | 9.85% | 159.8bp (149.7bp) | hsa-miR-342-3p MIMAT0000753 Homo sapiens miR-342-3p Targets (miRBase)(0.678) More Information | Similar Motifs Found | motif file (matrix) |
| 24 |  | 1e-202 | -4.652e+02 | 62.91% | 57.38% | 170.6bp (140.1bp) | hsa-miR-936 MIMAT0004979 Homo sapiens miR-936 Targets (miRBase)(0.698) More Information | Similar Motifs Found | motif file (matrix) |
| 25 |  | 1e-190 | -4.388e+02 | 24.22% | 19.73% | 172.3bp (141.8bp) | hsa-miR-4651 MIMAT0019715 Homo sapiens miR-4651 Targets (miRBase)(0.719) More Information | Similar Motifs Found | motif file (matrix) |
| 26 |  | 1e-182 | -4.197e+02 | 28.95% | 24.26% | 165.9bp (140.9bp) | hsa-miR-4485 MIMAT0019019 Homo sapiens miR-4485 Targets (miRBase)(0.648) More Information | Similar Motifs Found | motif file (matrix) |
| 27 |  | 1e-169 | -3.903e+02 | 27.00% | 22.58% | 170.8bp (140.0bp) | hsa-miR-675\* MIMAT0006790 Homo sapiens miR-675\* Targets (miRBase)(0.670) More Information | Similar Motifs Found | motif file (matrix) |
| 28 |  | 1e-154 | -3.552e+02 | 84.70% | 80.95% | 167.4bp (146.2bp) | hsa-miR-1305 MIMAT0005893 Homo sapiens miR-1305 Targets (miRBase)(0.798) More Information | Similar Motifs Found | motif file (matrix) |
| 29 |  | 1e-137 | -3.175e+02 | 52.71% | 48.07% | 160.7bp (147.1bp) | hsa-miR-551a MIMAT0003214 Homo sapiens miR-551a Targets (miRBase)(0.746) More Information | Similar Motifs Found | motif file (matrix) |
| 30 |  | 1e-130 | -3.008e+02 | 19.62% | 16.19% | 170.6bp (151.3bp) | hsa-miR-3186-5p MIMAT0015067 Homo sapiens miR-3186-5p Targets (miRBase)(0.656) More Information | Similar Motifs Found | motif file (matrix) |
| 31 |  | 1e-120 | -2.769e+02 | 10.90% | 8.39% | 171.5bp (145.5bp) | hsa-miR-4271 MIMAT0016901 Homo sapiens miR-4271 Targets (miRBase)(0.732) More Information | Similar Motifs Found | motif file (matrix) |
| 32 |  | 1e-107 | -2.480e+02 | 76.08% | 72.48% | 166.9bp (141.6bp) | hsa-miR-4500 MIMAT0019036 Homo sapiens miR-4500 Targets (miRBase)(0.762) More Information | Similar Motifs Found | motif file (matrix) |
| 33 |  | 1e-100 | -2.319e+02 | 1.38% | 0.64% | 139.2bp (146.0bp) | hsa-miR-32\* MIMAT0004505 Homo sapiens miR-32\* Targets (miRBase)(0.699) More Information | Similar Motifs Found | motif file (matrix) |
| 34 |  | 1e-39 | -9.189e+01 | 16.45% | 14.68% | 205.6bp (143.2bp) | hsa-miR-197 MIMAT0000227 Homo sapiens miR-197 Targets (miRBase)(0.678) More Information | Similar Motifs Found | motif file (matrix) |
